# Generalization of Psychosocial Skills to Life Skills in Collegiate Athletes

**DOI:** 10.3390/sports11020020

**Published:** 2023-01-17

**Authors:** Yuki Yabunaka, Ryo Kametani, Hironobu Tsuchiya

**Affiliations:** 1 Graduate School of Sport and Exercise Sciences, Osaka University of Health and Sport Science, 1-1 Asashirodai, Kumatori-cho, Sennan-gun, Osaka 590-0406, Japan; 2 Faculty of Commerce, University of Marketing and Distribution Sciences, 3-1 Gakuennishi-cho, Nishi-ku, Kobe-shi, Hyogo 651-2188, Japan; 3 School of Health and Sport Sciences, Osaka University of Health and Sport Sciences, 1-1 Asashirodai, Kumatori-cho, Sennan-gun, Osaka 590-0406, Japan

**Keywords:** basic need satisfaction/frustration, needs–supportive/thwarting behavior, structural equation modeling

## Abstract

This study investigated how needs–supportive and needs–thwarting coaching behaviors, basic need satisfaction (BNS), and basic need frustration (BNF) are positively or negatively related to collegiate athletes’ generalization of psychosocial skills in competitive sports. Japanese collegiate athletes (N = 228, *M*_age_ = 19.7 years) completed the measures to assess the study variables. Structural equation modeling for the hypothesized models of associations among the variables showed that needs–supportive coaching behavior positively influenced BNS, which, in turn, positively affected the generalization of all 10 psychosocial skills in competitive sports. Moreover, needs–thwarting coaching behavior had a positive influence on BNF, which, in turn, negatively affected the following skills: generalization of stress management, appreciating others, communication, and maintaining etiquette and manners. These findings highlight that coaches and life skills educational supporters should display needs–supportive behaviors that help to improve participants’ BNS and promote generalization of the psychosocial skills for life skills development through competitive sports. Furthermore, they should prevent needs–thwarting behaviors that frustrate their basic needs in competitive sports.

## 1. Introduction

Many studies have suggested that life skills can be developed and promoted through participation in competitive sports [1,2,3,4]. Life skills are personal and social competencies that serve as the foundation for psychosocial competencies that play an important role in the promotion of health in terms of physical, mental, and social well-being. It is defined as “abilities for adaptive and positive behavior that enable individuals to deal effectively with the demands and challenges of everyday life” [5] (p. 1). Shimamoto et al. [6] showed that the required life skills for Japanese athletes were derived from the actual experiences of top-level sports coaches, including, for example, stress management, setting goals, and thinking carefully.

The process of developing life skills through competitive sports is considered to generalize psychosocial skills to other life domains beyond competitive sports [7,8,9]. Psychosocial skills, as distinguished from life skills, are defined as abilities for adaptive and positive behavior that enable individuals to deal effectively with the demands and challenges of sports only [10]. Psychosocial skills in competitive sports are defined as abilities for adaptive and positive behavior that enable individuals to deal effectively with the demands and challenges of competitive sports [11]. Generalization is defined as the application of what is learned in a particular environment to other domains and environments [9]. Therefore, athletes who engage in competitive sports may effectively develop their life skills through the generalization of psychosocial skills in competitive sports.

However, Japanese collegiate athletes who belong to university athletic teams and devote themselves to competitive sports have pointed out various problems related to balancing academic and athletic activities [12], such as lower academic motivation [13], a decline in academic performance and an inability to fulfill graduation requirements due to an over-emphasis on athletic teams activities [14], and obtaining careers after university graduation, for instance, passive attitude for careers [15] and difficulties in obtaining a job due to time constraints caused by training, matches, and playing tours [16]. Previous research has found life skills are correlated to academic performance [17], career maturation [18], and career acquisition after graduation [16,19] among Japanese collegiate athletes. Considering the prior studies, these problems perhaps exist because they do not develop life skills effectively. Moreover, based on the process of developing life skills through competitive sports, since they may not be able to effectively generalize psychosocial skills in competitive sports to other life domains, they do not effectively develop life skills.

To solve these problems and enrich their college life and careers after college graduation, educational support to develop life skills for collegiate athletes is needed in Japan [20]. As collegiate athletes spend their student lives primarily focusing on competitive sports, effective support should encourage them to acquire life skills through it [21,22]. Therefore, the content of the support should be based on findings regarding the generalization of psychosocial skills while focusing on the process of development of life skills through competitive sports.

A theory that can be used to examine life skills development through sports is the life development intervention/basic needs theory model (LDI/BNT) [23]. Hodge et al. proposed that sport participants’ life skills are developed through satisfying the basic psychological needs in a needs–supportive motivational climate [24]. The LDI framework highlights the main outcome is fostering life skills, which can lead to increase personal competence and greater psychological functioning [25]. In addition, BNT, which is a sub-theory of self-determination theory (SDT), proposes that when the three basic needs of autonomy, competence, and relatedness are satisfied, people will experience positive psychological development and optimal psychological well-being [26]. Autonomy relates to individuals’ ability to make decisions and act in accordance with their sense of self. Competence refers to experiencing a sense of mastery through effective interactions within a social environment. Relatedness is defined as feelings of connection to and acceptance by others. The BNT model represents an increasing internalization of values, as one’s basic psychological needs are progressively satisfied, such that their behavior becomes internally regulated rather than primarily externally controlled [24]. Furthermore, when psychosocial skills values are central to an individual’s sense of self, those values are more likely to motivate psychosocial skills-related actions. Consequently, the more that individuals internalize the basic needs, the more likely they are to develop the ability to “generalize” psychosocial skills to a number of life contexts (e.g., school, family, part-time work, and job) [23]. The basic psychological needs are critically influenced by a needs–supportive motivational climate, which refers to the goals and behaviors emphasized with respect to the three basic needs and values that are salient in the social environment created by significant others [27]. On the basis of these tenets, Hodge et al. suggested a needs–supportive motivational climate created by significant others is positively associated with participant’s basic psychological needs, which, in turn, is positively associated with their life skills development. Previous research has supported the development of life skills in competitive sports through the process for Canadian youth sports participants at a competitive level by quantitative approach [28] and for former high school sports by qualitative approach [29]. Therefore, this model examines the process of life skills development through competitive sports.

In sports, in addition to basic need satisfaction (BNS), basic need frustration (BNF) has been examined. Vansteenkiste and Ryan suggested that BNS helps facilitate a person’s development, whereas BNF undermines it [30]. Supporting Vansteenkiste and Ryan’s idea, previous research found that athletes’ perceptions of BNS were related to vitality [31,32] and perceptions of continuing to win in proportion [33], whereas their experiences with BNF predicted emotional and physical exhaustion [31], burnout [32], and their moral disengagement in doping and attitudes toward the acceptance of cheating [33]. Therefore, based on the Hodge et al.’s propositions, the more that individuals frustrate the basic needs, the more likely they are to decline the ability to “generalize” psychosocial skills to a number of life contexts. Furthermore, BNS and BNF were proposed to be asymmetrical [30]. Warburton et al. outlined that physical education (PE) students and voluntary leisure-time sport participants reported more optimal outcomes when BNS was high and BNF was low, but less optimal outcomes when BNS was low and BNF was high [34]. Thus, it is postulated that BNS has a positive influence on the generalization of psychosocial skills in competitive sports, whereas BNF has a negative influence.

Furthermore, SDT postulates, either positively or negatively, that athletes need satisfaction and frustration. Specifically, when people in a sports context engage in needs–supportive interpersonal behaviors, they will promote the satisfaction of the basic psychological needs of athletes [35]. Alternatively, when people engage in (or are perceived to engage in) needs–thwarting interpersonal behaviors, athletes will experience need frustration [36]. SDT postulates six different types of interpersonal behaviors: autonomy-supportive, competence-supportive, relatedness-supportive, autonomy-thwarting (also called controlling), competence-thwarting, and relatedness-thwarting [37]. Previous research has reported that participants’ perception of coaches’ needs–supportive behaviors is positively correlated with BNS, although participants’ perception of coaches’ needs–thwarting behaviors is positively correlated with BNF for collegiate athletes [38], gym exercisers [39], and PE students [40]. The coach is a key agent of the social environment and can help either foster or forestall an athlete’s life skills development [8,41]. Thus, it is postulated that coaches’ needs–supportive and needs–thwarting behaviors correlate with collegiate athletes’ BNS and BNF in competitive sports.

Including both BNS and BNF and coaches’ needs–supportive and needs–thwarting behavior, Cronin et al. and Cronin, Ellison et al. investigated whether BNS mediated the association between autonomy-supportive behavior of coaches and sport participants’ learning of life skills for PE students [42] and participants in youth sports [43]. However, contrary to Cronin et al. and Cronin, Ellison et al., controlling teaching and coaching behaviors were not negatively related to participants’ life skills development in PE and youth sports [42,43].

Although these studies have contributed to the understanding of the mechanism of life skills development through sports, they have some limitations. First, this study investigated how coaches’ behavior and basic psychological needs influence life skills in sports. The process of developing life skills through competitive sports is considered to generalize psychosocial skills in competitive sports to other life domains beyond competitive sports [7,8,9]. Therefore, to better understand how collegiate athletes develop life skills in competitive sports, it is necessary to examine the influence of basic psychological needs on the generalization of psychosocial skills in competitive sports. Second, these studies concluded that BNF did not mediate any potential relationships between controlling teaching and participants’ development of life skills in PE and youth sports. Nonetheless, previous research found controlling coaching and BNF were negatively associated with athletes’ engagement [44] and mental toughness [45]. Therefore, it is assumed that the generalization of psychosocial skills in competitive sports could be affected not only by BNS but also by BNF. Finally, in these studies, only coaches’ autonomy-supportive and autonomy-thwarting (i.e., controlling coaching) behavior were investigated to examine whether basic psychological needs mediated the associations between coaches’ behavior and participants’ learning of life skills. SDT stipulates that supporting all three psychological needs, beyond just autonomy, should lead to an increase in needs satisfaction in athletes and a subsequent increase in athletes’ autonomous motivation for sports and other outcomes [46]. In addition to autonomy, research needs to examine competence and relatedness supportive/thwarting interpersonal behavior in coaches [38]. Therefore, the influence on collegiate athletes’ BNS or BNF in competitive sport from coaches’ needs–supportive/thwarting interpersonal behaviors including autonomy, competence, and relatedness supportive/thwarting behavior should be examined.

### The Current Study

Considering the studies highlighted above and expanding upon Cronin et al.’s and Cronin, Ellison et al.’s research [42,43], the current study sought to examine how coaches’ needs–supportive and –thwarting behaviors (i.e., competence and relatedness, in addition to autonomy bats), and BNS and BNF are either positively or negatively related to collegiate athletes’ generalization of psychosocial skills in competitive sports. This is a novel addition to the research literature, as few theory-based studies have investigated the process of life skills development through sports using a quantitative approach [8,23,47]. Focusing on the generalization of psychosocial skills in competitive sports, this study adds to our understanding of how exactly collegiate athletes develop their life skills within the context of competitive sports. Furthermore, this research provides valuable information for educational support to develop life skills through competitive sports for collegiate athletes.

**Figure 1:**
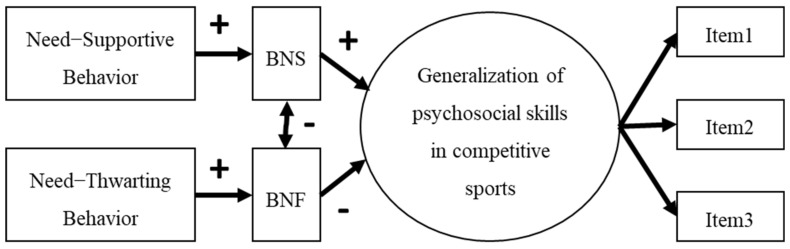
Hypothetical structural model of the associations between perception of need-supportive thwarting behavior, BNS, BNF, and generalization psychosocial skills in competitive sports.

The objective of this study was to examine the collegiate athletes’ generalization of psychosocial skills in competitive sports based on LDI/BNT [23] for life skills development through competitive sport through the demonstration of the following model (Figure 1). In line with the tenets of the LDI/BNT model [23], SDT [30], and past research findings [26,38,42,43] this model postulated that coaches’ needs–supportive behavior has a positive influence on collegiate athletes’ BNS, which, in turn, positively affected the generalization of psychosocial skills in competitive sports. In line with past studies on sports [44,45] and SDT-based propositions [29], we hypothesized that coaches’ needs–thwarting behavior has a positive influence on collegiate athletes’ BNF, which, in turn, negatively affected the generalization of psychosocial skills in competitive sports. Given that the BNS and BNF are proposed to be asymmetrical [30,34], it is assumed that there is a negative correlation between the BNS and BNF. In addition, Gould and Carson suggested that individual differences may affect life skills development in sports [48], and psychosocial skills in competitive sports generalization were correlated with collegiate athletes’ gender, grade, perception of similarity of actions between competitive sports and other life domains, and awareness of generalization possibilities between competitive sports and other life domains [11]. Thus, gender, grade, perception of similarity of actions, and awareness of generalization possibilities were controlled for in our hypothesized model analyses.

## 2. Materials and Methods

### 2.1. Participants

Participants included 296 Japanese undergraduates at two universities located in the Kinki region of Japan, 228 of whom were collegiate athletes (*M*_age_ = 19.7, *SD* = 1.1, range = 18–22 years) and comprised both males (*n* = 163) and females (*n* = 65) with participants having an average of 11.0 years (*SD* = 3.5) playing competitive sport experience.

The sports represented in the sample were soccer (*n* = 81), track and field (*n* = 21), baseball (*n* = 19), handball (*n* = 13), and soft tennis (*n* = 11). A small number of them participated in 25 other sports (e.g., volleyball, basketball, and badminton).

### 2.2. Measures

#### 2.2.1. Demographic Data

Demographic information including age, gender, type of sport, years of sports experience, highest grade in collegiate matches played, and number of days per week of activity of their current team was collected.

#### 2.2.2. Basic Needs Satisfaction and Frustration in Competitive Sport

The BNS and BNF in competitive sports were measured using the Japanese version of the Basic Psychological Need Satisfaction and Frustration Scale (BPNSFS) [49]. The BPNSFS consists of 12 need satisfaction items: four items for each basic psychological need (autonomy, relatedness, and competence) and 12 need-frustration items: four items for each basic psychological need. In the present study, following Nishimura and Suzuki [49], the factors of autonomy, relationship, and competence satisfaction were combined into the BNS, and the factors of each frustration were combined into the BNF. As this scale is not specific to competitive sports situations, following Toyama et al. [50], we asked participants to answer the extent to which each item applies to them in competitive sports situations. Participants rated each item on a five-point scale from 1 (completely disagree) to 5 (completely agree), indicating the extent to which their basic psychological needs were satisfied or unsatisfied in their competitive sport. Confirmatory factor analysis (CFA) supported a model that included two higher-order factors (need satisfaction and frustration) and six lower-order factors (autonomy, competence, and relatedness for satisfaction and frustration) [root mean square error of approximation (RMSEA) = 0.061, comparative fit index (CFI) = 0.877, Tucker–Lewis index (TLI) = 0.862, standardized root mean square residual (SRMR) = 0.081]. Cronbach’s alpha was 0.87 for the BNS and 0.87 for BNF in the current study.

#### 2.2.3. Coaches’ Needs–Supportive and –Thwarting Behaviors

The perceptions of interpersonal behaviors of coaches based on SDT within the context of needs–supportive and needs–thwarting behaviors were measured using the Japanese version of the Interpersonal Behaviors Questionnaire (IBQ-J) [51]. The IBQ-J consists of 12 needs–supportive behavior items: four items for each basic psychological need (autonomy, relatedness, and competence) and 12 needs–thwarting behavior items: four items for each basic psychological need. In the present study, to correspond to the factors of BNS and BNF in the BPNSFS, the factors of autonomy, relationship, and competence supportive behavior were combined into needs–supportive behavior, and the factors of each thwarting behavior were combined into needs–thwarting behavior. The item stem for each scale was “My coach …” following Rocchi et al. [38], and participants rated each item on a 7-point scale from 1 (completely disagree) to 7 (completely agree), indicating the extent to which interpersonal behaviors of coaches are consistent. CFA supported the factorial validity of a two-factor model, including needs–supportive and thwarting behaviors of coaches (RMSEA = 0.069, CFI = 0.867, TLI = 0.854, SRMR = 0.074). Cronbach’s alpha was 0.92 for needs–supportive behavior and 0.89 for needs–thwarting behavior in the current study.

#### 2.2.4. Generalization of Psychosocial Skills in Competitive Sports

The generalization of psychosocial skills in competitive sports to other life domains was measured using the Generalization Scale of Psychosocial Skills for Collegiate Athletes (GSPS-CA) [11]. The GSPS-CA consists of 30 items based on 10 dimensions of the required life skills for collegiate athletes [6]: with three items for each generalization of psychosocial skills in competitive sports. The participants rated each item on a 5-point scale from 1 (completely disagree) to 5 (strongly agree), indicating the extent to which psychosocial skills in competitive sports are generalized to other life domains (study, career selection, relationships, and everyday life). Cronbach’s alphas ranged from 0.59 to 0.86 for each dimension in the current study.

#### 2.2.5. Perception of Similarity of Action and Awareness of Generalization Possibilities between Competitive Sport and Other Life Domains

Each perception of similarity of action and awareness of generalization possibilities between competitive sports and other life domains were measured using the following questions [11]: ‘To what extent do you feel that the actions in competitive sports are similar to those in other life domains?’ and ‘To what extent do you feel that your experience in competitive sports is useful beyond competitive sports?’ The participants rated each item on a five-point scale from 1 (completely disagree) to 5 (strongly agree).

### 2.3. Data Analysis

For our preliminary analysis, correlations, and descriptive statistics, we used SPSS version 27.0. To examine whether the data supported the proposed model, we performed structural equation modeling (SEM) using Mplus 8.4. The following indices were used to evaluate the model fit: RMSEA, CFI, TLI, and SRMR. A good fit of the model to the data is characterized by an RMSEA smaller than 0.06 [52], CFI and TLI values larger than 0.90 [53], and an SRMR smaller than 0.08 [52].

### 2.4. Procedure

Approval was obtained from the ethics committee of the first author’s affiliated university before conducting the study (approval number = 21–29). The survey was conducted by central location test at the beginning of physical education classes and meetings for athletic club activities at each university. The purpose of the study and ethical considerations for the survey were explained both orally and in writing by the first author. The ethical considerations included the following: the survey results would be statistically processed and would not identify any individual, and the individual would be free to withdraw from the study at any given time without prejudice. Subsequently, participants were asked to sign an informed consent form if they agreed to participate. None of the participants chose to withdraw their participation. Furthermore, based on Yabunaka et al. [11], when participants answered the questionnaire, we emphasized that responses should indicate how true each statement was based on what they learned because of participation in competitive sports and not because of what they learned in other activities; that they should answer each item imagining the domain of generalization; and that given the possibility of social desirability response bias in the responses derived from the content of the instructional text, responses should be provided honestly. Participants took roughly 10–15 min to complete the survey.

## 3. Results

### 3.1. Preliminary Analyses

Gould and Carson suggested that individual differences may affect the development of sports life skills [50]. Furthermore, gender and grade differences, the degree of similarity of action, and awareness of generalization possibilities were correlated with the generalization of psychosocial skills in competitive sports for collegiate athletes [11]. Therefore, gender, grade, degree of similarity of action, and awareness of generalization possibilities differences were assessed across all variables in the hypothesized models. Regarding gender, the t-test showed that females scored significantly higher than males on their ratings of appreciating others (*t* (226) = −2.35, *p* < 0.05) and taking responsibility for one’s own behavior (*t* (226) = −1.95, *p* < 0.05). For grade, ANOVA showed that first-year college students scored significantly lower than third-year students on their ratings of stress management (*F* (2, 215) = 3.69, *p* < 0.05), and first-year college students scored significantly higher than second year college students’ ratings of perceived needs–supportive coaching behavior (*F* (2, 215) = 4.18, *p* < 0.05). For similarity of action, ANOVA showed that there were statistically significant differences in all variables’ scores (*F* (2, 217) = 3.20–17.40, *p* < 0.05). For awareness of generalization possibilities, ANOVA showed that there were statistically significant differences in all variables’ score except for setting goals (*F* (2, 210) = 3.65–9.87, *p* < 0.05). Based on the above results, we controlled for these four factors by including them as covariates in our SEM.

### 3.2. Descriptive Statistics

The means and standard deviations for each variable along with bivariate correlations are presented in Table 1. The associations between needs–supportive behavior and participants’ BNS (*r* = 0.46) and the generalization of each psychosocial skills in competitive sports (*r* range = 0.14–0.35) were positive and significant. The BNS was positively and significantly associated with the generalization of each psychosocial skills in competitive sports (*r* range = 0.34–0.61). The associations between needs–thwarting behavior and participants’ BNF (*r* = 0.63) and generalization of each psychosocial skills in competitive sports (*r* range = 0.14–0.25), except for setting goals, thinking carefully, and maintaining physical health and well-being, were negative and significant. BNF was negatively and significantly associated with generalization of each psychosocial skills in competitive sports except for setting goals and thinking carefully (*r* range = −0.21–0.40). Given that there were no statistically significant associations between needs–thwarting behavior, BNF, and generalization of the three psychosocial skills in competitive sports, SEM for the hypothesized models of setting goals, thinking carefully, and maintaining physical health and well-being were conducted, except for needs–thwarting behavior and BNF, and these were entered as covariates in all models.

### 3.3. SEM for the Hypothesized Models

**Figure 2:**
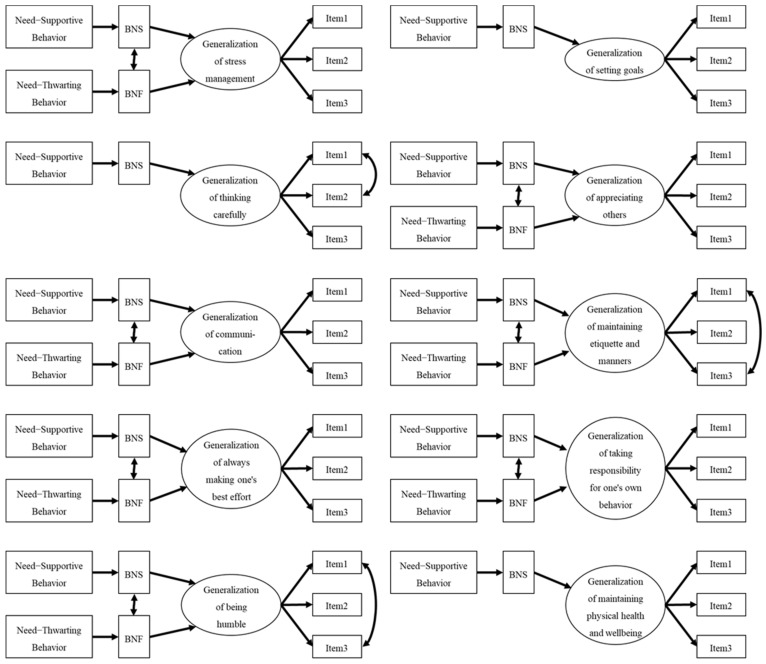
The final SEM models of associations between perception of needs–supportive/thwarting behavior, BNS, BNF, and generalization of psychosocial skills in competitive sports.

SEM initially revealed the fit indices of each hypothesized model (as shown in Figure 1), except for setting goals, thinking carefully, and maintaining physical health and well-being in our data (Table 2). The fit indices of each model of stress management, appreciating others, communication, always making one’s best effort, and taking responsibility for one’s own behavior were as follows: RMSEA = 0.059–0.065, CFI = 0.954–0.971, TLI = 0.907–0.942, and SRMR = 0.046–0.052. RMSEA of several models were > 0.06, however CFI, TLI, and SRMR were good fit. Thus, a mostly adequate fit is shown in these models (Figure 2). However, given that the fit indices of the model of maintaining etiquette and manners were RMSEA = 0.078, CFI = 0.933, TLI = 0.866, and SRMR = 0.053, and being humble were RMSEA = 0.077, CFI = 0.934, TLI = 0.868, and SRMR = 0.055, inadequate fits were shown in the two models. The modification indices suggested by Mplus advised the addition of correlation between each item of maintaining etiquette and manners and being humble. The modified models (Figure 2) were a mostly adequate fit to the data, maintaining etiquette and manners were RMSEA = 0.065, CFI = 0.956, TLI = 0.908, and SRMR = 0.048, and being humble were RMSEA = 0.053, CFI = 0.970, TLI = 0.936, and SRMR = 0.046.

Furthermore, the fit indices of each hypothesized model of setting goals, thinking carefully, and maintaining physical health and well-being were tested (Table 2). The fit indices of each goal model were RMSEA = 0.059, CFI = 0.953, TLI = 0.901, and SRMR = 0.063, and maintaining physical health and well-being were RMSEA = 0.032, CFI = 0.983, TLI = 0.963, and SRMR = 0.036. Thus, an adequate fit was shown in the two models (Figure 2). However, given that the fit indices of the model of thinking carefully were RMSEA = 0.078, CFI = 0.933, TLI = 0.866, and SRMR = 0.053, an inadequate fit was shown in the model. The modification indices suggested by Mplus advised the careful addition of correlations between items of thinking carefully. The modified models (Figure 2) adequately fit the data (RMSEA = 0.046, CFI = 0.972, TLI = 0.937, SRMR = 0.056).

Subsequently, we tested the relationship between the variables in each hypothetical model (Table 2). In the models of stress management, appreciating others, communication, maintaining etiquette and manners, always making one’s best effort, taking responsibility for one’s own behavior, and being humble, needs–supportive behaviors positively affected BNS (*β* = 0.44, *p* < 0.01), and needs–thwarting behaviors positively affected BNF (*β* = 0.55, *p* < 0.01). The generalization of these psychosocial skills in competitive sports were significantly positively influenced by the BNS (*β* range = 0.26–0.73, *p* < 0.01). Stress management, appreciating others, communication, and maintaining etiquette and manners were significantly negatively influenced by BNF (*β* range = 0.17–0.23, *p* < 0.05, or *p* < 0.01). A significant positive association was found between BNS and BNF (*r* = 0.36, *p* < 0.01). The proportions of variance explained by each model were as follows: BNF (21%), BNS (38%), and generalization of each psychosocial skills in competitive sports (20–63%). In the models of setting goals, thinking carefully, and maintaining physical health and well-being, needs–supportive behaviors positively affected the BNS (*β* = 0.43, *p* < 0.01). The generalization of these psychosocial skills in competitive sports was significantly positively influenced by the BNS (*β* range = 0.36–0.66, *p* < 0.01). The proportions of variance explained by each model were as follows: BNS (21%), BNF (38%), and generalization of each psychosocial skills in competitive sports (30–52%).

## 4. Discussion

This study aimed to examine collegiate athletes’ generalization of psychosocial skills in competitive sports based on LDI/BNT for life skills development through competitive sport. SEM for the hypothesized models showed that coaches’ needs–supportive behavior had a positive influence on collegiate athletes’ BNS, which, in turn, positively affected the generalization of the 10 psychosocial skills in competitive sports. Moreover, coaches’ needs–thwarting behavior had a positive influence on collegiate athletes’ BNF, which, in turn, negatively affected the generalization of stress management, appreciating others, communication, and maintaining etiquette and manners in the analyses. Previous studies have focused on life skills development [42,43], while the current study examined the process of life skills development through competitive sports, focusing on the generalization of psychosocial skills in competitive sports [7,8,9]. These results, focusing on the generalization of psychosocial skills in competitive sports based on LDI/BNT, provide a better understanding of the process of life skills development through sports.

In the current study, we demonstrated that coaches’ needs–supportive/thwarting behavior, including autonomy, competence, and relatedness, influenced collegiate athletes’ perceptions of BNS and BNF in competitive sports. While Rocchi et al. suggested that research should examine all three needs–supportive/thwarting interpersonal behaviors in coaches [38], previous research [42,43] focused only on the autonomy-supportive and autonomy-thwarting behavior of coaches. Thus, our findings provide more information on life skills coaching in competitive sports.

Our results on correlations between coaches’ needs–supportive behavior, total need satisfaction, and generalization of psychosocial skills in competitive sports were consistent with the insights of Bean et al. [28], Cronin et al. [42], and Cronin, Ellison et al. [43] that BNS mediated the associations between autonomy-supportive coaching and youth sports and PE participants’ development of life skills. Few quantitative theory-based studies have investigated the development of life skills [8,23,47]. Our findings support Hodge et al.’s [23] conceptual model for life skills development and their proposition by a quantitative approach and suggest that LDI/BNT and SDT (i.e., coaches’ needs–supportive behavior and BNS) can serve as a theoretical framework for further investigating life skills development in competitive sports. This also supports Deci and Ryan’s idea that a combination or balance of all three basic needs is needed for positive psychological development [54].

Contrary to the results of Cronin et al. [42] and Cronin, Ellison et al. [43], the present study showed that needs–thwarting behavior was correlated with BNF and had statistically significant associations with participants’ generalization of some psychosocial skills in competitive sports. These results were consistent with previous studies in sports showing that controlling coaching and BNF are negatively associated with other positive outcomes, such as an athlete’s engagement [44] and mental toughness [45]. Furthermore, our findings support Vansteenkiste and Ryan’s [30] SDT-based proposition that BNF can partially undermine a person’s development. It is possible that BNF affected the generalization of stress management, appreciating others, communication, and maintaining etiquette and manners for the following reasons. Japanese collegiate athletes live student lives, mainly focusing on competitive sports. Therefore, it is likely that BNF in competitive sports has a significant influence on BNF in their student lives beyond competitive sports. Given that all three BNS were positively correlated with participants’ perceived good relationships with friends, teachers, and their class [55], BNF is probably negatively associated with participants’ perceived relationships with them for collegiate athletes. In addition, these four skills are important for developing good relationships with others. From the above, BNF in student life influenced by BNF in competitive sports may have collegiate athletes perceive fewer good relationships with others beyond competitive sports. Therefore, it is likely that they are prevented from performing these four skills in student life, beyond competitive sports.

### 4.1. Practical Implications

This study provides valuable information for educational support to develop life skills through competitive sports for collegiate athletes. An online survey of official sports coaches certified by the Japan Sports Association has revealed that inappropriate behavior in sports coaching (i.e., controlling coaching, for example, verbal abuse, corporal punishment/hazing, sexual harassment) is still presented in some situations [56]. Therefore, The Japan Sports Association has supported official sport instructors with the skills to promote appropriate sporting activities for people at every stage of life [57]. In terms of supporting coaches, our findings highlighted that exhibiting needs–supportive behaviors and avoiding needs–thwarting behaviors are important skills for coaches to display to develop collegiate athletes’ life skills through generalization of psychosocial skills in competitive sports. In this regard, for example, Rocchi et al. [38] presented that autonomy-supportive behavior supports athletes’ decisions, competence-supportive behavior is telling them that they can accomplish things, and relatedness-supportive behavior is taking the time to get to know them. Moreover, Rocchi et al. [38] indicated that autonomy-thwarting behavior imposes coaches’ opinions on them, competence-thwarting behavior sends them the message that they are incompetent, and relatedness-thwarting behavior is distant when they spend time together [38]. Based on our findings, such needs–supportive coaching behaviors promote collegiate athletes’ need satisfaction for autonomy, competence, and relatedness and, in turn, help them generalize their psychosocial skills in competitive sports. On the other hand, such needs–thwarting coaching behaviors improve collegiate athletes’ needs frustration, in turn preventing them from generalizing their psychosocial skills in competitive sports. To translate our LDI/BNT-based findings into practice, formal training of coaches in life skills development is required [58], and life skills programs should educate instructors and coaches about the benefits of using LDI/BNT-based principles in practice.

In terms of supporting collegiate athletes, our findings highlighted that satisfying basic psychological needs and preventing the frustration associated with these needs in competitive sports are valuable educational support for developing collegiate athletes’ life skills through the generalization of psychosocial skills in competitive sports. In terms of the need for autonomy, it could be the effective support that they acquire the skills related to autonomy (e.g., independent thinking, maintaining physical health and wellbeing, stress management) and utilize them in the context of competitive sports. Regarding the need for competence, supporters of life skills development should teach them goal-setting skills (e.g., setting short- and long-term goals) that are related to competence in competitive sports. In terms of the need for relatedness, team-building programs in their team could be introduced to create positive relationships among collegiate athletes, coaches, and peers that ensure they understand and trust each other. Collegiate athletes can learn skills associated with relatedness (e.g., appreciating others, communication, and maintaining etiquette and manners) through such activities.

### 4.2. Limitations and Future Directions

The first limitation of this study was that the collected data could be affected by both social desirability and memory recall [59]. Hence, in future research, coaches’ needs–supportive/thwarting behavior and collegiate athletes’ generalization of psychosocial skills in competitive sports should be assessed by other social agents in their student lives. A second limitation is that we adopted a cross-sectional design in this study; thus, causality between the variables could not be examined. For this reason, future studies should use longitudinal research designs to investigate participants’ learning of life skills in competitive sports through the generalization of psychosocial skills in competitive sports. A third limitation is that the needs–supportive/thwarting behavior of social agents other than coaches was not assessed in this study. Mossman et al. [60] demonstrated that coaches, as well as parents and peers, have a positive influence on youth sports participants’ life skills development. Therefore, future research could investigate whether the needs–supportive/thwarting behavior of their parents and peers has an effect on collegiate athletes’ basic psychological needs and generalization of psychosocial skills in competitive sports. Fourth, this study only focused on total need satisfaction and frustration due to examine the associations among coaches’ need supportive/thwarting behavior, BNS and BNF. Hodge et al. [23] postulated that life skills development occurs via the satisfaction of each of the three basic needs associated with skills. Future studies could investigate whether each of the three needs satisfaction and frustration influence the generalization of each psychosocial skills in competitive sports associated with needs. A fifth limitation is that this study did not consider the individual pre-existing makeup and personality traits of the collegiate athletes. Camiré et al. suggested that their pre-existing makeup and personality traits play a part in their life skills development [61]. Thus, future research could investigate if collegiate athletes’ pre-existing makeup and personality traits moderate the effects of coaches’ needs–supportive and –thwarting behaviors and their generalization of psychosocial skills in competitive sport.

## 5. Conclusions

Based on the LDI/BNT model [23], the novel findings from the current study showed that needs–supportive coaching is positively related to BNS, which, in turn, positively affects collegiate athletes’ generalization of psychosocial skills in competitive sports [23]. Furthermore, needs–thwarting coaching is positively related to BNF, which, in turn, negatively affects collegiate athletes’ generalization of some psychosocial skills in competitive sports. These results highlight that coaches and life skills educational supporters seeking to foster participants’ life skills development through competitive sports should aim to create a needs–supportive climate that satisfies participants’ three basic psychological needs and break a needs–thwarting climate that frustrates their three needs in practice.

## Figures and Tables

**Table 1:** Descriptive statistics and bivariate correlations.

		*M*	*SD*	1	2	3	4
Interpersonal behaviors of coaches							
1.	Needs–supportive behaviors	4.71	1.01				
2.	Needs–thwarting behaviors	3.26	1.05	−0.54 **	-		
Basic Psychological Need in competitive sport							
3.	BNS	3.70	0.56	0.46 **	−0.34 **	-	
4.	BNF	2.33	0.68	−0.21 **	0.63 **	−0.45 **	-
Generalization of psychosocial skills in competitive sports							
5.	Stress management	3.80	0.81	0.14 *	−0.14 *	0.34 **	−0.28 **
6.	Setting goals	3.07	0.85	0.27 **	−0.02	0.39 **	0.00
7.	Thinking carefully	3.56	0.70	0.18 **	0.02	0.48 **	−0.13
8.	Appreciating others	4.21	0.68	0.19 **	−0.19 **	0.52 **	−0.35 **
9.	Communication	4.03	0.64	0.20 **	−0.21 **	0.50 **	−0.39 **
10.	Maintaining etiquette and manners	4.16	0.58	0.23 **	−0.25 **	0.50 **	−0.40 **
11.	Always making one’s best effort	3.91	0.63	0.35 **	−0.20 **	0.61 **	−0.32 **
12.	Taking responsibility for one’s own behavior	3.91	0.63	0.29 **	−0.19 **	0.59 **	−0.29 **
13.	Being humble	3.84	0.66	0.26 **	−0.19 **	0.49 **	−0.29 **
14.	Maintaining physical health and wellbeing	3.68	0.78	0.27 **	−0.08	0.38 **	−0.21 **

* *p* < 0.05, ** *p* < 0.01.

**Table 2:** Results of SEM for each generalization of psychosocial skills in competitive sports.

		*χ* ^2^	*df*	RMSEA	CFI	TLI	SRMR	BNS Generalization of PS-CS	BNF Generalization of PS-CS	The Proportions of Variance of Generalization of PS-CS
1.	Stress management	35.70	20	0.059	0.971	0.942	0.046	0.26 **	−0.19 **	0.20
2.	Setting goals	28.86	16	0.059	0.953	0.901	0.051	0.48 **		0.30
3.	Thinking carefully	22.38	15	0.046	0.972	0.937	0.047	0.66 **		0.52
4.	Appreciating others	39.24	20	0.065	0.963	0.926	0.052	0.46 **	−0.17 *	0.39
5.	Communication	34.97	20	0.057	0.966	0.933	0.049	0.45 **	−0.23 **	0.43
6.	Maintaining etiquette and manners	37.21	19	0.065	0.956	0.908	0.048	0.52 **	−0.22 **	0.55
7.	Always making one’s best effort	39.41	20	0.065	0.954	0.907	0.049	0.73 **	−0.05	0.63
8.	Taking responsibility for one’s own behavior	37.83	20	0.063	0.961	0.922	0.050	0.65 **	−0.04	0.53
9.	Being humble	31.38	19	0.053	0.970	0.936	0.046	0.55 **	−0.13	0.47
10.	Maintaining physical health and wellbeing	19.83	16	0.032	0.983	0.963	0.036	0.36 **		0.37

* *p* < 0.05, ** *p* < 0.01.

## Data Availability

All relevant data are within the manuscript. The datasets generated during and/or analyzed during the current study are available from the corresponding author upon reasonable request.
